# FUT8-Mediated Core Fucosylation Promotes the Pulmonary Vascular Remodeling in Pulmonary Arterial Hypertension

**DOI:** 10.14336/AD.2023.0218

**Published:** 2023-10-01

**Authors:** Wen Zhang, Wenchao Lin, Xiaofang Zeng, Mengqiu Zhang, Qin Chen, Yiyang Tang, Jing Sun, Benhui Liang, Lihuang Zha, Zaixin Yu

**Affiliations:** ^1^Department of Cardiology, Xiangya Hospital, Central South University, Changsha, Hunan, China; ^2^Department of nephrology, Xiangya Hospital, Central South University, Changsha, Hunan, China; ^3^National Clinical Research Center for Geriatric Disorders (Xiang Ya), Changsha, Hunan, China

**Keywords:** pulmonary arterial hypertension, pulmonary vascular remodeling, core fucosylation, FUT8

## Abstract

Pulmonary arterial hypertension (PAH) is a progressive cardiopulmonary disease with unclear underlying molecular mechanisms and limited therapeutic options. This study aimed to explore the role of core fucosylation and the only glycosyltransferase FUT8 in PAH. We observed increased core fucosylation in a monocrotaline (MCT)-induced PAH rat model and isolated rat pulmonary artery smooth muscle cells (PASMCs) treated with platelet-derived growth factor-BB (PDGF-BB). We found that 2-fluorofucose (2FF), a drug used to inhibit core fucosylation, improved hemodynamics and pulmonary vascular remodeling in MCT-induced PAH rats. In vitro, 2FF effectively restrains the proliferation, migration, and phenotypic switching of PASMCs and promotes apoptosis. Compared with controls, serum FUT8 concentration in PAH patients and MCT-induced rats was significantly elevated. FUT8 expression appeared increased in the lung tissues of PAH rats, and the co-localization of FUT8 with α-SMA was also observed. SiRNA was used to knockdown FUT8 in PASMCs (siFUT8). After effectively silencing FUT8 expression, phenotypic changes induced in PASMCs by PDGF-BB stimulation were alleviated. FUT8 activated the AKT pathway, while the admission of AKT activator SC79 could partially counteract the negative effect of siFUT8 on the proliferation, apoptotic resistance, and phenotypic switching of PASMCs, which may be involved in the core fucosylation of vascular endothelial growth factor receptor (VEGFR). Our research confirmed the critical role of FUT8 and its mediated core fucosylation in pulmonary vascular remodeling in PAH, providing a potential novel therapeutic target for PAH.

## INTRODUCTION

Pulmonary hypertension (PH) is a clinical and pathophysiological syndrome characterized by elevated pulmonary artery pressure after abnormalities of structure or dysfunction of pulmonary vessels, leading to right heart failure and even death. Pulmonary arterial hypertension (PAH), a major subcategory of PH, is considered at a mean pulmonary artery pressure (mPAP) ≥ 20 mmHg, pulmonary artery wedge pressure (PAWP) ≤ 15 mmHg, and pulmonary vascular resistance (PVR) > 2WU at rest [1]. PAH is a common condition with a prevalence of 15-100 cases/million adults, and its prognosis is unfavorable with poor quality of life and high mortality [2]. Pulmonary vascular remodeling is the main pathological feature of PAH, and primarily involves pulmonary arterioles [3, 4]. Commonly used targeted therapeutic drugs aim to alleviate pulmonary vasodilation. Although these drugs can alleviate the symptoms of patients with PAH and improve the survival rate to a certain extent, the long-term prognosis of PAH is still not optimistic if the pulmonary vascular remodeling cannot be reversed [5]. Therefore, the underlying mechanism of pulmonary vascular remodeling requires further investigation to better understand the pathogenesis of PAH, determine novel therapeutic targets, and improve patient prognosis.

Glycosylation is one of the most important types of post-translational modifications. Protein glycosylation refers to the transfer of monosaccharides or glycans to a specific binding site on a protein through the action of glycosyltransferase, which can alter the stability and subcellular localization and affect the function of the modified protein [6]. After the receptor proteins are modified by glycosylation, the ligand and receptor interaction and the intracellular signal transduction can be changed, further affecting cell proliferation, apoptosis, migration, and other pathophysiological processes [7, 8]. Alterations in glycosylation levels are common and currently observed in various diseases, such as diabetes, gastrointestinal diseases, and breast cancer [9, 10].

According to the type of monosaccharide or glycan, protein glycosylation can be divided into different categories, including fucosylation, mannitylation, and saliva acidification. Core fucosylation is a special form of fucosylation mediated by FUT8 in the Golgi apparatus and involves binding a fucose to the N-acetyl glucosamine group of the protein via α-1,6-glycoside bonds. FUT8-mediated core fucosylation plays an important role in the proliferation and invasion of various cancer cells, including liver cancer, breast cancer, melanoma, pancreatic cancer, endometrial cancer, and lung cancer [11-20]. However, the effects of core fucosylation and FUT8 on cardiovascular disease are unclear. Furthermore, the relationship among core fucosylation, FUT8, and PAH has not been explored.

Considering that PASMCs have many tumor-like characteristics, such as disordered proliferation and resistance to apoptosis, the present study aimed to explore the role of FUT8-mediated core fucosylation in the pathogenesis of PAH. The study aims to provide new ideas to explore the underlying mechanism of PAH, as well as aid in the development of diagnostic and therapeutic options.

## MATERIALS AND METHODS

### PAH animal model

Healthy male Sprague-Dawley (SD) rats weighing approximately 180 g were obtained from the SLAC Laboratory Animal Company. All rats were housed in specific pathogen-free conditions in the Animal Facility of the Central South University Animal Center (Changsha China), in an animal room with a 12 h light-dark cycle at a temperature of 20-25 °C with 40-60% humidity. All procedures involving rats and all experimental protocols were approved by the Central South University Animal Care and Use Committee (No. 2021sydw0091). Treatment-group rats were injected with a dose of 60 mg/kg MCT (Sigma, C2401, and dissolved in physiological saline, 5mg/ml) intraperitoneally to establish a PAH model, and control model rats were injected with the same amount of physiological saline. For the 2FF treatment group, 75 mg/kg 2FF (Selleck, S9954, and dissolved in physiological saline, 25 mg/ml) was intraperitoneally injected on alternating days from the third day after MCT injection. We recorded weight changes and the survival rate of rats. An assessment of PAH and a histological analysis were both made on the 21^st^ day after MCT injection.

### Hemodynamics and right ventricular remodeling

After the rats were anesthetized with 1% pentobarbital (4 mL/kg) intraperitoneal injection, the right ventricular systolic pressure (RVSP) and mean pulmonary arterial pressure (mPAP) were recorded by right catheterization. After hemodynamics data were detected, serum, heart, and lung tissue samples were collected. The right ventricle (RV) and left ventricle plus interventricular septum (LV + S) were separated and weighed separately to calculate the RV/(LV + S) mass ratio.

### HE staining and immunofluorescence

In order to observe the process of vascular morphology, we utilized HE staining. The tissue slices were kept in xylene solution for 30 min then put in 100% ethanol solution, 95% ethanol solution, and 70% ethanol solution in that order for 5 min each for dewaxing rehydration. The samples were then washed in deuterieum-depleted water and stained with hematoxylin, 1% hydrochloride ethanol differentiation. After this, the samples were stained with eosin and then put in 95% ethanol to remove excess pigment followed by 100% ethanol and xylene for 10 min to dehydrate them prior to sealing.

Next, we used immunofluorescence to observe vascular morphology and molecular localization. Here, the samples were maintained in 80 °C sodium citrate repair antigen solution for 20 min and hydrogen peroxide for 10 min at room temperature after dewaxing rehydration, following the method used for HE staining. Samples were then washed thrice with PBS. Next, the samples were allowed to permeate and were pre-incubated with 3% BSA solution with 0.3% Triton100 at room temperature for 1 h. α-SMA (1:200, Proteintech, 67735-1-Ig) or FUT8 (1:100, Bioss, bs-24389R) primary antibodies or 20 μg∕mL biotinylated lectins (Vectorlabs, B-1045) were then used prior to incubating samples at 4 °C overnight, followed by incubation with Alexa Fluor 594-AffiniPure Goat Anti-Rabbit IgG (1:200, Jackson, 111-585-144), Alexa Fluor 594-labeled goat anti-mouse IgG (1:200, Thermo Fisher Scientific, A-11005), Alexa Fluor 488-AffiniPure Goat Anti-Mouse IgG (1:200, Jackson, 115-545-166), or DTAF-conjugated Streptavidin (1:100, Jackson, 016-010-084) for 1 h at room temperature in the dark. The negative control group was incubated with primary antibody diluent at 4 °C overnight followed by the same fluorescent secondary antibodies as other groups. Finally, the cell nuclei were stained with DAPI (Beyotime Biotechnology, China) for 10 min at room temperature, and images were captured with a fluorescence microscope after sealing.

### Isolation of pulmonary artery smooth muscle cells (PASMCs) and cell culture

To explore the effect of FUT8 and its mediated core fucosylation on our PAH cell model, we extracted rat PASMCs for analysis. Normal male SD rats weighing approximately 200 g were selected then euthanized after 1% pentobarbital anesthesia and sterilized with 70% ethanol. Next, cardiopulmonary tissue was placed into 20% FBS (Gibco, Invitrogen, Burlington, Canada) medium and transferred into a sterile primary cell desk. We then separated the pulmonary artery from the bottom of the heart in as long a piece as possible, removed the surrounding connective tissue, cut the vessel along the long axis with ophthalmic scissors, and scraped off the inner and outer membranes gently with blunt forceps. The artery was cut into tissue blocks of approximately 1 mm^2^ in size, dispersed, and attached to the bottom of the dispersion. When the tissue blocks were dried slightly, F12/DMEM medium supplemented with 20% FBS and 1% antibiotic/antimycotic (Gibco) was added along the side wall. Tissue blocks were then cultured in a primary cell culture incubator, changing the medium once after four to five days. When the cells migrated out of the tissue pieces and fused, they were then digested and rested for 30 minutes. We retained the adherent cells (differential adhesion) for subsequent culture experiments.

The PASMCs of the 3rd-6th generation were then grown in F_12_ /DMEM medium supplemented with 10% FBS, 1% antibiotic/antimycotic and 1% smooth muscle cell growth supplement (Sciencell, #1152). Platelet-derived growth factor BB (PDGF-BB, 30 ng/mL, PeproTech, 100-14B) was used to establish PAH-PASMCs, and 2-fluorofucose (2FF, 100 μmol/L, Cayman, 17171) was used to reduce the overall core fucosylation levels of the PASMCs.

### Cell proliferation assays

To test cell proliferation, the culture supernatant was discarded and replaced with Cell Counting Kit-8 (CCK8) solution (NCM Biotech, China). The experiment was conducted in accordance with the product instructions, and the cells were incubated at 37 °C with 5% CO_2_ for 2 h after adding medium with CCK8. Absorbance values (OD) were determined using a microplate reader at a wavelength of 450 nm.

### Immunoprecipitation

The cells were cultured in 10 cm dishes, proteins were extracted with 600 μl NP40 lysis buffer (Epizyme, Shanghai), with protease inhibitor and phosphatase inhibitor cocktails (Roche, Basel, Switzerland), and centrifuged to obtain the supernatant. Primary antibody against VEGFR (1:100, Santa Cruz, sc-393179) was used to bind to the protein at 4 °C overnight. Subsequently, 6 μl proteinA agarose beads (Roche, 11719408001) were added to the samples after washing twice and resuspending samples in 60 μl NP40. The samples were then mixed well by swirling them for 3 h at 4 °C, followed by centrifugation at 2,000 rpm for 2 min to collect the agarose beads. Thereafter, beads with proteins were washed twice, centrifuged, and boiled to use for subsequent experiments.

### Western blotting and lectin blotting

For western blotting and lectin blotting analysis, protein lysates were separated on reducing SDS-polyacrylamide gel electrophoresis (PAGE) gels at 120 V for approximately 60 min, and transferred to polyvinylidene fluoride (PVDF) membranes at 300 mA for 90 min. The membranes were then blocked with 5% skim milk or 3% BSA for 1.5 h at room temperature, and probed with primary antibodies against VEGFR (1:500, Santa cruz, sc-393179), FUT8 (1:1000, Bioss, bs-24389R), p-AKT (1:1000, Cell Signaling, 4060T), AKT (1:1000, Proteintech, 10176-2-AP), β-actin (1:2000, Proteintech, 20536-1-AP), PCNA (1:2500, Proteintech, 60097-1-Ig), OPN (1:1000, Wanleibio, WL00691), BCL-2 (1:1500, Proteintech, 12789-1-AP), and BAX (1:2000, Proteintech, 50599-2-Ig) at 4 °C overnight on a shaker. Next, membranes were incubated with HRP-conjugated AffiniPure goat anti-rabbit (1:4000, Proteintech, SA00001-2) or anti-mouse (1:3000, Proteintech, SA00001-1) IgG for 1 h at room temperature after washing with TBST. For lectin blotting, the membranes were blocked with 3% BSA overnight at 4 °C and incubated with 2 μg∕mL biotinylated lectins (Vector Laboratories, United States) for 2 h at room temperature, followed by incubation with HRP-conjugated Avidin (1:3000, Boster, BA1088) for 1 h at room temperature. Bands of target molecules were detected using an ECL system and the gray values were quantified using ImageJ software.

**Figure 1.**
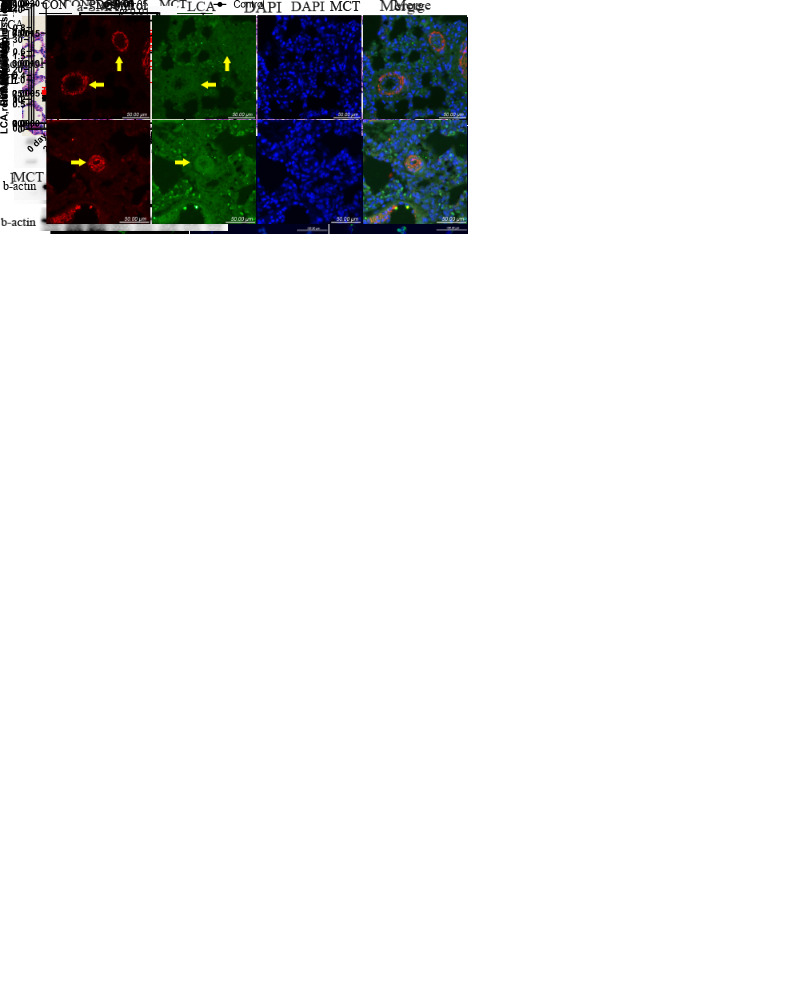
Core fucosylation is upregulated in MCT-induced PAH rat lung tissues and PASMCs stimulated with PDGF. RVSP of rats in control (N=8) and MCT group (N=8) detected by right heart catheterization is shown in (A) and mPAP of rats in control (n=5) and MCT group (n=5) is shown in (B). Weight changes of rats in control (n=5) and MCT (n=8) groups are shown in (C). Right heart hypertrophy index (D) and right heart weight ratio (E) of rats in control (n=5) and MCT groups (n=6) were measured. (F) HE staining of pulmonary tissues is shown and the center of the views indicate arterioles. (G) Core fucosylation of homogenous proteins in control and MCT-rat lung tissues was measured using lectin blotting (n=4). β-actin was used as a housekeeping protein for normalization. Quantification of core fucosylation level is shown. (H) Immunofluorescence of rat lung tissue slice is shown, and the arrow indicates pulmonary arterioles. (I) Core fucosylation level of cell proteins in control and PDGF-PASMCs was measured using LCA blotting (n=5). (J) Immunofluorescence of cells in control and PDGF stimulating groups is shown. All results are shown as mean ± SD. Unpaired t-tests were performed for comparisons of means between two groups in (A), and Mann-Whitney U tests were performed for the data in other charts. The MCT group was compared with the control group. Among them, p-values are indicated above the groups being compared with each other. RV indicates right ventricle; RVSP indicates right ventricular systolic pressure; mPAP indicates mean pulmonary artery pressure; LV+S indicates left ventricle plus ventricular septum; BW indicates body weight; MCT indicates monocrotaline; LCA indicates lens culinaris agglutinin.

### RNA isolation and quantitative RT-PCR

To measure FUT8 expression in the lung tissue, we extracted total mRNA from the MCT and the control rats using TRIzol. Tissue blocks were lysed in 1 mL TRIzol and ground with a low temperature high speed tissue homogenizer at 60 Hz for 180 s. Subsequently, 200 μl chloroform was used for phase separation, and 100% isopropanol was used for RNA precipitation. Finally, the RNA was dissolved in 30 μl DEPC water after being washed with 80% ethanol. FUT8 expression was detected after the RNA was reverse transcribed using the cDNA Synthesis Kit and BlazeTaq™ SYBR Green qPCR Mix (GeneCopoeia), according to the manufacturer’s instructions. *β-actin* was used as a housekeeping gene for quantitative RT-PCR. The following primers were used for *FUT8*: sense, 5′-GCTACCGATGACCCTGCTTTG-3′; antisense, 5′-GCTACCGATGACCCTGCTTTG-3′, and *β-actin*: sense, 5′-GGAGATTACTGCCCTGGC TC CTA-3′; antisense, 5′-GGAGATTACTGCCCTGGC TC CTA-3′.

### siRNA transfection

To perform siRNA transfection, the cells were first seeded into six-well plates. Transfection was then performed using a transfection kit (Ribo) according to the manufacturer’s instructions with 100 μM siRNA. siRNA was also synthesized by the Ribo biological company, and the oligonucleotide sequences of siRNAs were: (1) siNC 5′-TTCTCCGAACGTGTCACGTdTdT-3′; (2) siFUT8 5′-CCAGCGGAGAAUAACUUAUTTAUAAGUUAU UCUCCGCUGGTT-3′; (3) siVEGFR2 5′-CCTCAAAG CAT CAGCATAA-3′.

### Flow cytometry analysis

Suspension cells and adherent cells were collected in six-well plates in order to detect the level of apoptosis using the Annexin V-FITC/PI apoptosis assay kit (A211-01, Vazyme, China) according to the manufacturer’s instructions. The apoptosis rate was quantified using FlowJo software (Q2+Q3).

### Cell scratch wound assay

To conduct our cell scratch wound assay, cells were uniformly seeded in a 12-well plate, cultured in a 37 °C cell culture incubator, transfected, and dosed with PDGF-BB, 2FF, or siRNA. We quickly and gently created a scratch in the culture using a 200 μl pipette tip, washed the cells twice with PBS, and replaced the medium with DMEM/F12 with 0.5% FBS. Images of cell migration in the same position were photographed at 0 h and 24 h after the scratch. The wound healing by migrating cells was compared to the total area of the scratch, for both the treatment and control groups using ImageJ.

### ELISA testing

A Human FUT8 ELISA Kit (EK1990, Boster Biological Technology Company) and an ELISA Kit for rats (FY40556, FeiYa Biotechnology) were used to detect the concentration of FUT8 according to the manufacturer’s instructions. Plasma and serum samples were collected, diluted, incubated in microplate-coated plates, washed, microplate labeled, and color rendered sequentially according to the manufacturer’s instructions. A microplate reader was used to detect the absorbance of each well at 450 nm. From this we constructed a standard sample concentration-absorbance curve and calculated the FUT8 concentration of the samples according to the absorbance of each well.

### Statistical methods

Using GraphPad Prism 8 software for statistical analysis and plotting. The Shapiro-Wilk normality test was performed to assess the normality of the distribution of data. The comparison of the means of two groups was conducted using unpaired, 2-tailed Student’s *t* tests or Mann-Whitney U tests for normally and nonnormally distributed data, respectively. To calculate the comparisons between multiple groups (≥3 groups), ANOVA was performed. *p*<0.05 was used to indicate a statistically significant test result. All data were replicated with three or more biological samples and expressed as the mean ± standard deviation. Other statistical details are reported in the figure legends.

**Figure 2.**
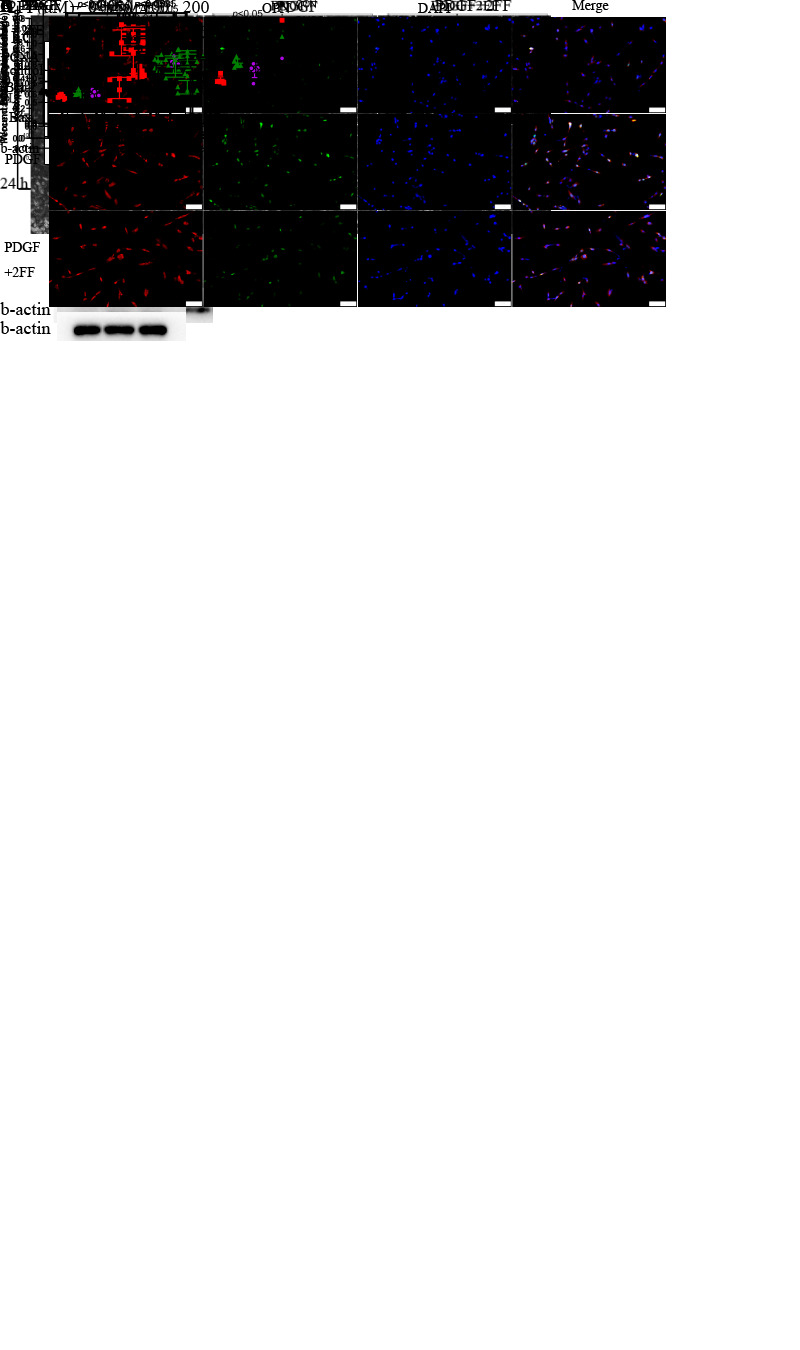
Core fucosylation promotes the proliferation, migration, and apoptosis resistance of PASMCs. (A) Effects of various concentrations of 2FF on core fucosylation of PASMC proteins after 48 h are shown. (B) Effects of 100 μM 2FF on the core fucosylation of proteins in PASMCs and quantitative analysis (at upper right corner, n=5) are shown. A time point of 48 h and a concentration of 100 μM were chosen for use in subsequent experiments. Proliferative activity of PASMCs treated with PDGF or 2FF detected using the CCK8 assay (n=6) is shown in (C). (D) Protein expression levels of PCNA (n=6) and Bcl-2/Bax (n=5) in control and PDGF-PASMC groups treated with or without 2FF were measured using western blotting. β-actin was used as a housekeeping protein for normalization. Quantifications of protein level are shown. (E) The phenotypic conversion of PASMCs to a synthetic type was detected using immunofluorescence, Scale bar=100 μm. (F) Quantification of apoptotic rate of PASMCs was detected using flow cytometry (n=5). (G) Effect of 2FF on migration capacity of PASMCs was determined using the scratch experiment (n=4), Scale bar=100 μm. All results are shown as mean ± SD. Data were analyzed by one way ANOVA. PDGF group was compared with the control group and 2FF-treated group separately. p-values are indicated above the groups being compared with each other. 2FF indicates 2-deoxy-2-fluoro L-Fucose. Ns indicates no statisticallysignificant.

## RESULTS

### Core fucosylation is upregulated in MCT-induced PAH rat lung tissue and PASMCs stimulated with PDGF.

The established MCT-induced PAH rat model was evaluated via right heart catheterization and HE staining. The group of PAH rats displayed higher RVSP (mean, control: 22.21 mmHg, MCT: 47.34 mmHg, Fig. 1A), mPAP (mean, control: 12.05 mmHg, MCT: 20.97 mmHg, Fig. 1B), and right heart hypertrophy index (mean, control: 0.21, MCT: 0.65, Fig. 1D), as well as thicker media layers, and greater inflammatory cell infiltration compared with the control group (Fig. 1F). The core fucosylation levels in PAH were assessed via immunofluorescence staining and lectin blotting. As a type of lectin, LCA can bind to core fucose specifically. Therefore, the level of core fucosylation was upregulated in the lung tissues of MCT-induced PAH rats compared with controls (Fig. 1G), and increased core fucosylation was mainly observed in smooth muscle cells, as illustrated by the co-expression of α-SMA staining (Fig. 1H). PDGF (30 ng/mL) was used *in vitro* to stimulate PASMCs for 24 h to establish the PAH cell model. Consistently, the level of core fucosylation of PASMCs increased significantly after PDGF stimulation compared with control cells (Fig. 1I-J).

### Core fucosylation promotes proliferation, migration, and apoptosis resistance of PASMCs

After determining the upregulated core fucosylation in the PAH model, we next aimed to explore whether increased core fucosylation may play a role in PAH progression rather than its accompanying symptoms. 2FF was applied into core fucosylation inhibition, and we found that a dose of 100 μM could significantly attenuate core fucosylation of the whole cell protein by approximately 70% (Fig. 2A-B). Compared to the group treated with only PDGF-BB, the group with added 2FF displayed lower proliferative activity, as shown using the CCK8 assay (Figure 2C), and expression of PCNA, a marker of the proliferative phenotype, was also decreased by 2FF (Fig. 2D). In addition, 2FF inhibited the apoptotic resistance of PASMCs treated with PDGF-BB. The proportion of apoptotic cells (Q2 and Q3 in flow cytometry, Fig. 2F) was significantly reduced after 2FF treatment, and the apoptotic resistance marker BCL-2/BAX ratio appeared decreased (Fig. 2D). We found that 2FF can ameliorate phenotypic switching from contractile to synthetic, as immunofluorescence showed that the fluorescence intensity of OPN decreased (Fig. 2E). Besides, 2FF decreased the scratch healing speed of PASMCs, suggesting it could reduce their migration capacity (Fig. 2G).

### 2FF improved hemodynamics and pulmonary vascular remodeling in MCT-induced PAH rats

**Figure 3.**
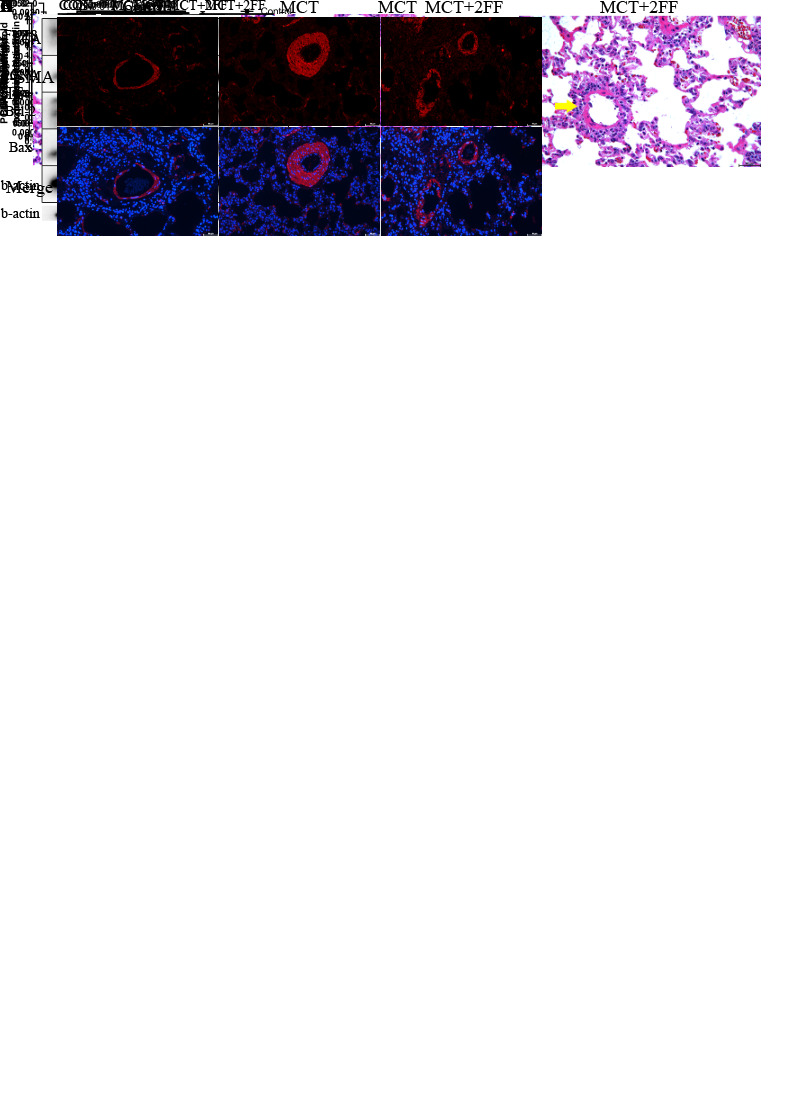
2FF improved the hemodynamics and pulmonary vascular remodeling in MCT-induced PAH rats. Weight changes of rats in control (N=4), MCT and 2FF treatment groups (N=6) are shown in (A), and RVSP of rats detected by right heart catheterization is shown in (B). Right heart hypertrophy index (C) and right heart weight ratio (D) of rats in the three groups were measured. Immunofluorescence (E) and HE staining (F) were used to evaluate the arterial media thickness and the arrow indicates the pulmonary arterioles. Scale bar=50 μm. (G) Protein expression levels of FUT8, PCNA, Bcl-2/Bax in control (n=4), MCT and 2FF treatment groups (n=6) were measured using western blotting. β-actin was used as a housekeeper protein for normalization. Quantifications of the protein levels are shown. (H) Core fucosylation levels of homogenous proteins in control (n=4), MCT and 2FF treatment-rat (n=6) lung tissues were measured using lectin blotting, and β-actin was used as a housekeeper protein for normalization. Quantification of core fucosylation level is shown. All results are shown as mean ± SD. Data were analyzed by one way ANOVA. MCT group was compared with the control group and 2FF treatment group separately. p-values are indicated above the groups being compared with each other. Ns indicates no statistically significant.

We explored whether the inhibition of core fucosylation has an efficient therapeutic effect *in vivo*. In comparison with the negative control group, the rats injected intraperitoneally with MCT had a relatively lower body weight, and the combination of 2FF with MCT showed a similar weight-gain curve as those treated with MCT alone (Fig. 3A). 2FF can effectively ameliorate the MCT-induced elevation of RVSP (mean, MCT: 47.18 mmHg, 2FF: 29.74 mmHg, Fig. 3B) and right heart hypertrophy index (mean: MCT: 0.47, 2FF: 0.29, Fig. 3C). HE and immunofluorescence staining results of α-SMA indicated that pulmonary vascular remodeling can be relieved with 2FF (Fig. 3D-F). Next, lung homogenate protein was extracted. Results show that 2FF reduced the level of core fucosylation *in vivo* (Fig. 3H), and the expression of PCNA was reduced (Fig. 3G). However, the ratio of Bcl-2/Bax showed no significant difference due to the number of samples and lung tissue interference. Interestingly, 2FF reduced the expression of FUT8 in lung tissues.

**Figure 4.**
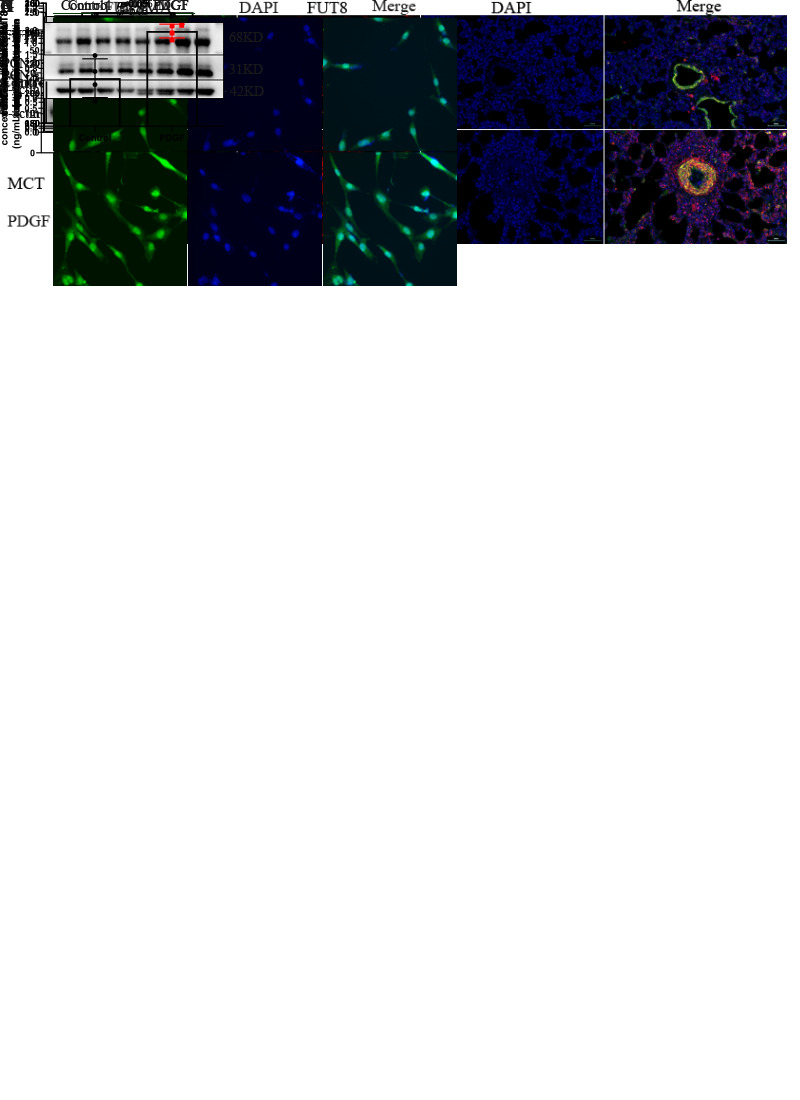
FUT8 was upregulated in both PAH patients and our rat experimental model. Concentrations of FUT8 in human (A) and rat (B-C) blood samples were detected by ELISA. There were 11 volunteers, 11 patients with CTD-PAH or IPAH, and 13 patients with CHD-PAH (diagnosed PAH by right heart catheterization in Xiangya Hospital). The blood samlpes of rats were from 8 control rats and 11 rats injected MCT. (D) Immunofluorescence of α-SMA and FUT8 in rat lung tissue slice is shown, Scale bar=50 μm. (E) Protein expression levels of FUT8 and PCNA in control and MCT-rat lung tissue homogenate were measured using western blotting (n=4). β-actin was used as a housekeeper protein for normalization. Quantifications of the protein levels are shown. (F) mRNA exprssion of FUT8 in rat lung tissue homogenization was determined by qPCR (n=4). (G) Protein expression levels of FUT8 (n=4) and PCNA (n=5) in control and PDGF-PASMCs were measured useing western blotting. (H) Immunofluorescence of FUT8 in control and PDGF-PASMCs is shown. All results are shown as mean ± SD. Data of three groups in (A) were analyzed by one way ANOVA, and the Mann-Whitney U test was performed for the comparisons between control and MCT (or PDGF) groups in other charts. p-values are indicated above the groups being compared with each other.

**Figure 5.**
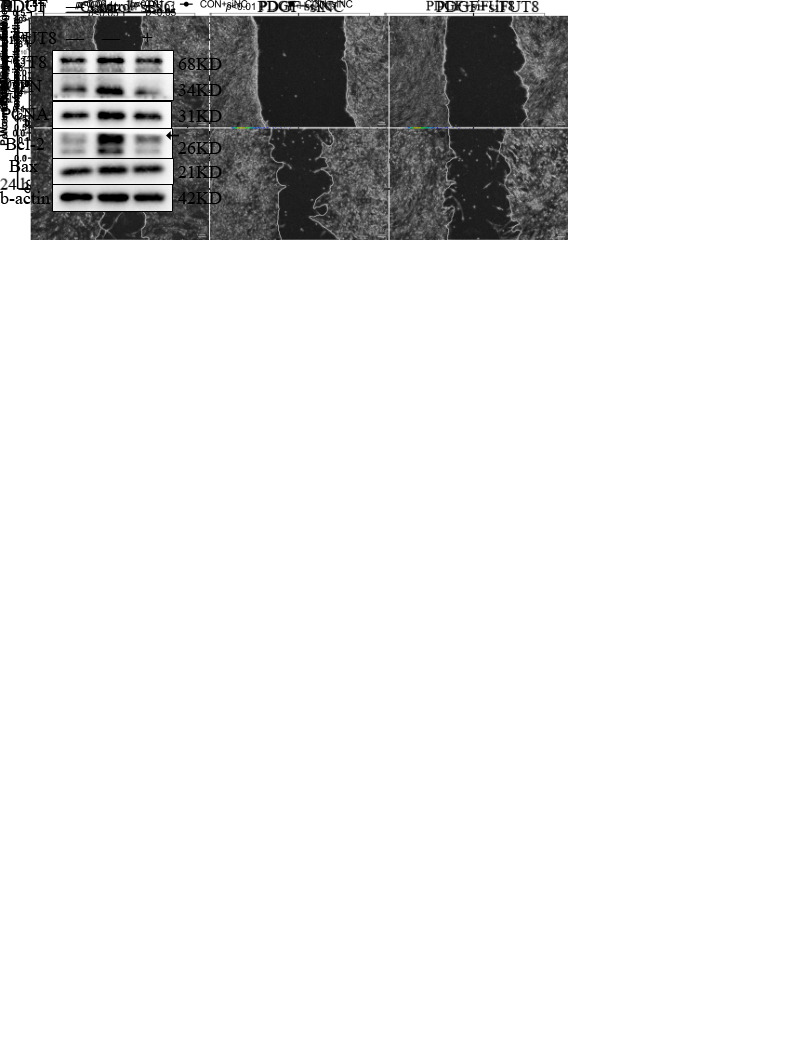
FUT8 silencing impairs proliferation, migration, apoptosis resistance, and phenotype conversion of PASMCs. (A) Proliferative activity of PASMCs treated with PDGF or siRNA was detected using the CCK8 assay (n=6). (B) Protein expression levels of FUT8 (n=4), PCNA (n=4), Bcl-2/Bax (n=4), OPN (n=5) in control and PDGF-PASMCs treated with or without knocking down FUT8 were measured using Western blotting. β-actin was used as a housekeeper protein for normalization. Quantifications of protein levels are shown. (C) Apoptotic rate of PASMCs-knocked down FUT8 was detemined using flow cytometry (n=5). (D) Effect of knocking down FUT8 on migration capacity of PASMCs was determined using the scratch experiment (n=4), Scale bar=100 μm. All results are shown as mean ± SD. Data were analyzed by one way ANOVA. PDGF+siNC vs. control+siNC group; PDGF+siFUT8 vs. PDGF+siNC group. p-values are indicated above the groups being compared with each other.

**Figure 6.**
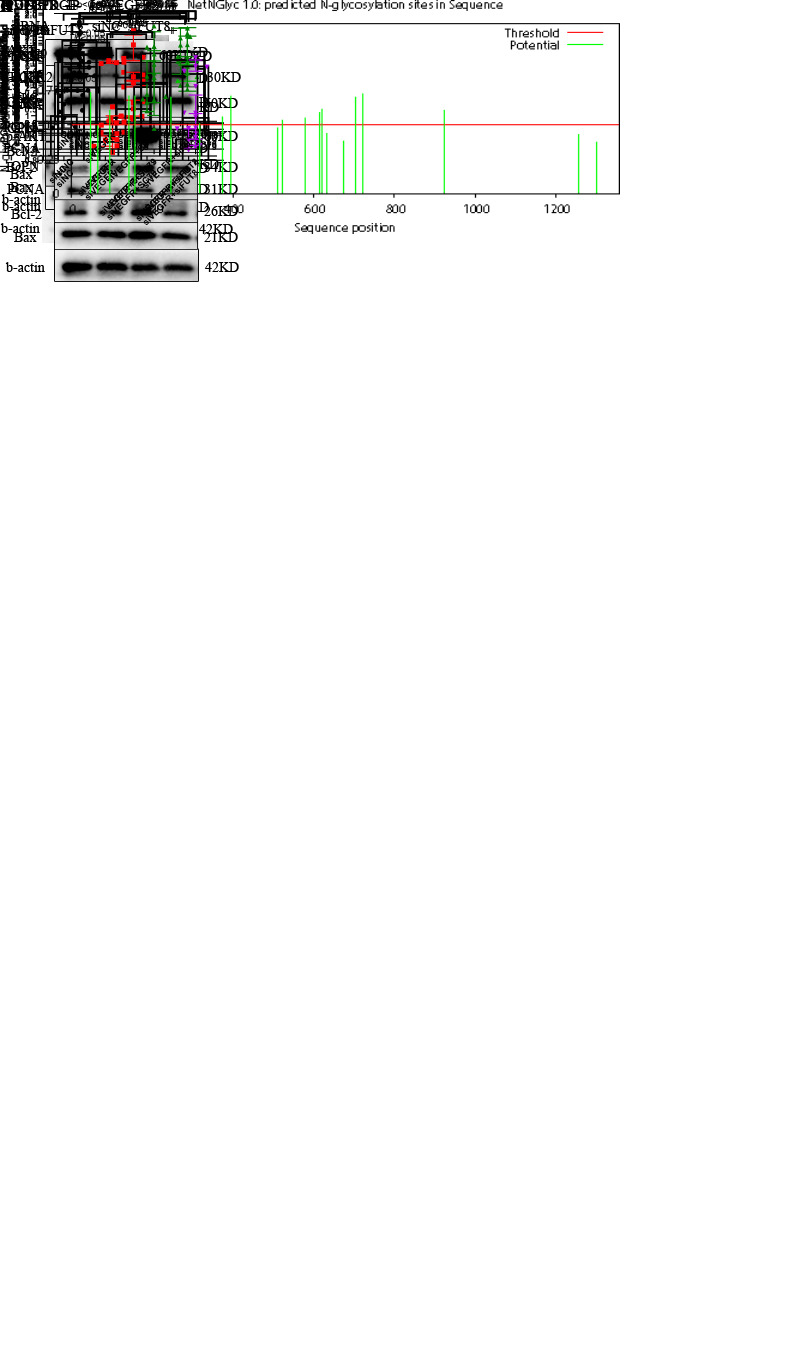
FUT8 regulates the core fucosylation of VEGFR to activate the AKT pathway. Effect of siFUT8 on AKT pathway (A) and rescure experiment (B) were detemined using western blotting (n=4). Protein expression levels of PCNA, OPN, p-AKT/AKT, and Bcl-2/Bax were quantificated and β-actin was used as a housekeeper protein for normalization. (C) N-glycosylation sites for VEGFR were dredicted by NetNGlyc1.0 Server online. (D) Effect of knocking down both VEGFR and FUT8 on the AKT pathway was detemined using western blotting. Protein expression levels of PCNA, OPN, p-AKT/AKT, and Bcl-2/Bax were quantificated (n=4). β-actin was used as a housekeeper protein for normalization. (E) Core fucosylation levels of cell proteins in control and PDGF-PASMCs after knocking down FUT8 were measured using lectin blot (n=4). β-actin was used as a housekeeper protein for normalization. Quantification of the core fucosylation level is shown. (F) Core fucosylation and protein expression level of VEGFR2 in immunoprecipitated proteins were measured using lectin blot and western blotting. All results are shown as mean ± SD. Data were analyzed by one way ANOVA. p-values are indicated above the groups being compared with each other.

### FUT8 was upregulated in both PAH patients and our rat experimental model.

FUT8 is the only known enzyme responsible for α-1,6 fucosylation, which is also known as core fucosylation. Core fucosylation displayed a relationship with PAH. Therefore, the role of FUT8 in PAH progression was determined. Blood samples were collected from patients with PAH and healthy donors, and FUT8 concentration was measured using ELISAs. In comparison with controls, serum FUT8 concentration was significantly increased in patients with PAH, including idiopathic PAH, connective tissue-associated PAH, and congenital heart disease-associated PAH (Fig. 4A). The FUT8 concentration in the serum and plasma of PAH rats was then determined. Results show that the concentration was significantly elevated compared with the control group (Fig. 4B-C). Immunofluorescence of lung tissue slices showed that FUT8 expression increased in the middle layer of the pulmonary arteries in the PAH group, with higher fluorescence intensity and overlapped with α-SMA (Fig. 4D). Furthermore, proteins and RNA were extracted from lung tissue homogenates, and the expression of FUT8 was elevated in the PAH model based on western blot analysis and qRT-PCR results (Fig. 4E-F). *In vitro*, PDGF-BB stimulation also upregulated FUT8 expression (Fig. 4G-H).

### FUT8 silencing impairs proliferation, migration, apoptosis resistance, and phenotypic switching of PASMCs.

The role of FUT8 in PAH was explored using siRNA to silence the expression of FUT8. PASMCs were transfected with siRNA targeting FUT8 (siFUT8) or negative control (siNC). siFUT8 can effectively inhibit FUT8 expression. In addition, silencing FUT8 expression could inhibit excessive proliferation (Fig. 5A-B), apoptotic resistance (Fig. 5B-C), and phenotypic switching (Fig. 5B) of PASMCs induced by PDGF-BB. The scratch experiment indicated that PASMCs transfected with siFUT8 showed reduced migration capability compared with these transfected with siNC (Fig. 5D).

### FUT8 regulates the core fucosylation of VEGFR to activate the AKT pathway.

The specific mechanism underlying FUT8-mediated phenotypic changes to PASMCs remains unclear. The AKT signaling pathway is over-activated in PAH and is closely associated with the proliferation and apoptosis resistance of PASMCs [21, 22]. The influence of FUT8 knockdown on the activation status of the AKT pathway was determined. Results showed that FUT8 silencing could reduce the levels of phosphorylated AKT (Fig. 6A). Besides, the admission of SC79 (an AKT activator) could partially counteract the negative effect of siFUT8 on the proliferation, apoptotic resistance, and phenotypic switching of PASMCs (Fig. 6B). As a highly glycosylated protein (Fig. 6C), VEGFR can activate the AKT pathway [23-25]. Therefore, VEGFR silencing can inhibit AKT activation, proliferation, and promote apoptosis of PASMCs, which can be reversed by SC79 (Fig. 6D). Moreover, the level of core fucosylation of the overall cytoplasmic proteins (Fig. 6E) and core fucosylated VEGFR (Fig. 6F) decreased significantly after FUT8 silencing. Meanwhile, VEGFR2 and FUT8 were silenced, and the effects of their silencing did not appear to be additive (Fig. 6D). The results suggest that FUT8 did not function when VEGFR was silenced, and VEGFR may be a downstream effector of FUT8.

## DISCUSSION

Vascular remodeling of the pulmonary artery is an important pathological feature of PAH. Pulmonary outer membrane fibroblasts, the extracellular matrix, and inflammatory cells are involved in this progress, but the thickening of the middle membrane caused by the excessive proliferation and apoptosis resistance of PASMCs is the most important factor leading to pulmonary vascular remodeling [26-30]. Accordingly, scholars have studied PASMCs by exploring the effect of traditional Chinese medicine [31-36], and the roles of molecules related to tumors and other diseases [37-40], as well as the effect of noncoding RNAs, such as microRNAs and LncRNAs on PASMC phenotypic changes [41-48].

Core fucosylation and the expression of FUT8 were upregulated in our PAH animal and cell models. Reducing core fucosylation or downregulating the expression of FUT8 could ameliorate vascular remodeling and the phenotypic changes of PASMCs, such as proliferation and apoptosis resistance. By exploring the effect of FUT8 and its mediated core fucosylation on PASMCs, this experiment links glycosylation with pulmonary hypertension to provide new ideas for exploring the mechanism underlying pulmonary hypertension development, and sheds light on new ideas for clinical treatment options.

Several studies have explored the relationship between glycosylation and cardiovascular disease. For example, N-glycosylation is the most common glycosylation modification in eukaryotic cells, and changes in N-glycan modifications on the cell surface of endothelial or immune cells and proteins associated with these cells affect cell ion channel function, inflammatory response, and lipid metabolism, thus influencing the cardiovascular system [49, 50]. The enzyme α-mannitosidase can affect N-glycan modification on the surface of vascular endothelial cells and thus affect the recruitment and adhesion of inflammatory cells [51]. Moreover, stachytine hydrochloride (an active ingredient in motherwort granules) can increase the number and N-glycosylation level of β1 adrenergic receptors to activate the cAMP/PKA signaling pathway and improve myocardial function [52].

Another major type of glycosylation modification, O-glycosylation, refers to the binding of acetamidogalactose (GalNAc) or acetylglucose (GlcNAc) to the hydroxyl(-OH) group of the polypeptide containing serine (Ser) and threonine (Thr). O-GlcNAc modification is also involved in the regulation of various signaling pathways within cells, thus regulating growth, proliferation, hormone response, and other processes, and playing an important role in neurodegenerative diseases, diabetes, and other metabolic diseases [53, 54]. O-GlcNAc glycosylation levels have been regulated in transgenic mice by overexpressing O-GlcNAc transferase (OGT) or its hydrolases (OGA). Increased O-GlcNAc glycosylation can affect myocardial energy metabolism, promote myocardial cell hypertrophy, aggravate ventricular remodeling, and lead to heart failure [55]. PAH can eventually lead to right heart failure and even death. If myocardial function can be improved by regulating glycosylation to compensate for the increased pulmonary circulation pressure, it may serve as a new idea for the treatment of pulmonary hypertension. Several studies have also explored the relationship between pulmonary hypertension and glycosylation and have shown that the level of N-acetyl glucosamine (GlcNAc) is increased in idiopathic pulmonary hypertension. Moreover, OGT promotes the proliferation of PASMCs and the expression of vascular endothelial growth factor (VEGF). Furthermore, OGT knockdown or the use of its inhibitors can inhibit the proliferation of PASMCs and angiogenesis [56, 57].

As a special class of N-glycosylation, core fucosylation primarily mediates cell adhesion and immune killing in tumors and immune diseases [58-60]. Increased core fucosylation, along with an increase in salivary acidification and branch glycan structure, is the most frequently occurring cancer-related change among protein glycosylation [10]. The interaction of FUT8 with the proliferation and migration of various tumor cells and its epithelial-mesenchymal transition (EMT) have been studied [61-63]. In some cancers, FUT8 expression is significantly higher than in paracancerous tissues or cells and affects cell invasion, metastasis, and drug resistance [12, 17, 64]. In addition, highly expressed FUT8 can increase the susceptibility of cells to viruses and maintain a long-term state of infection [65, 66]. Our experimental results show that the elevated expression level of FUT8 promotes the switching of PASMCs from a contractile phenotype to a synthetic phenotype with high proliferation and migration capacities but low apoptosis percentage, which is consistent with the effects of FUT8 and its mediated core fucosylation in tumor cells reported in previous research. Furthermore, FUT8 regulates pericyte activation through multiple signaling pathways, transforms pericytes into myofibroblasts, and promotes pulmonary fibrosis [67-69]. Pulmonary fibrosis is a lung disease leading to group 3 pulmonary hypertension.

To further explore the specific mechanism of action of FUT8 on the phenotypic changes in smooth muscle cells, we examined possible protein receptors and signaling pathways. Most protein receptors undergo glycosylation, which affects the receptor function. Various receptors, such as TGF-βR, PDGFR, and EGFR, have core fucosylation modifications. Increasing core fucosylation activates downstream signaling pathways, such as the Smad2/3 and ERK pathways. Most of these studies targeted animal models with peritoneal fibrosis, renal interstitial fibrosis, or pulmonary fibrosis and found that inhibiting FUT8 expression inhibited disease progression [68-73]. Limited studies have focused on the role of FUT8 in the cardiovascular system, but only one study has demonstrated that FUT8 regulates the TGF-β/Smad2/3 signaling pathway in the aortic smooth muscle of a uremia rat model by influencing core fucosylation of TGF-βR II and ALK5, resulting in vascular calcification [74].

Thus, the specific effect of core fucosylation mediated by FUT8 on protein function needs to be determined. FUT8 knockout causes reduced core fucosylation in fibroblasts and affects the binding of EGFR to its ligands, thus affecting the activation of downstream ERK and JNK pathways [75]. Moreover, increasing sialic acidification or α-1,3 fucosylation mediated by FUT4/FUT6 of EGFR in lung cancer cells inhibits its dimerization and phosphorylation, and reducing core fucosylation mediated by FUT8 induces the same effects and inhibits the activation of various receptors and downstream pathways [76].

Based on the role of FUT8 and core fucosylation in PASMCs, we focused on the AKT pathway, a signaling pathway affecting proliferation and migration in cells, and selected upstream molecules that may be core fucosylated. VEGFR is a highly N-glycosylated protein, and studies have shown that VEGFR plays a role in proliferation and migration of vascular smooth muscle cells [77-79]. Glycosylation modification sites of VEGFR exist in both its extracellular ligand binding domain and the domain associated with intracellular receptor activation. Highly processed glycans, including those for salivary acidification and fucosylation, are present at the site named N145 [80]. In addition, the level and type of N-glycosylation on VEGFR regulate ligand-dependent receptor activation. For example, sialic acidification at the N247 site inhibits receptor activation, and N-glycosylation deletion caused by mutations at this site promotes receptor dimer formation and phosphorylation activation [81]. However, limited studies have focused on the effect of core fucosylation on VEGFR activation and downstream pathway activation. In this study, the presence of core fucosylation modifications on the VEGFR-2 by immunoprecipitation was demonstrated, and the results show that reducing the level of core fucosylation affected its downstream AKT pathway, providing a new idea for the progress and treatment of pulmonary hypertension by influencing glycosylation levels.

However, this experiment has some limitations. Our follow-up work intends to reduce the expression of FUT8 in animal models to determine its role *in vivo*. In terms of mechanism exploration, specific core fucosylation sites on VEGFR are still sought, and the effect of core fucosylation on receptors can be effectively demonstrated by point mutations and rescue experimentation. Meanwhile, more information about proteins with altered core fucosylation levels will be obtained by lectin pull-down experiments combined with mass spectrometry.

**Figure 7.**
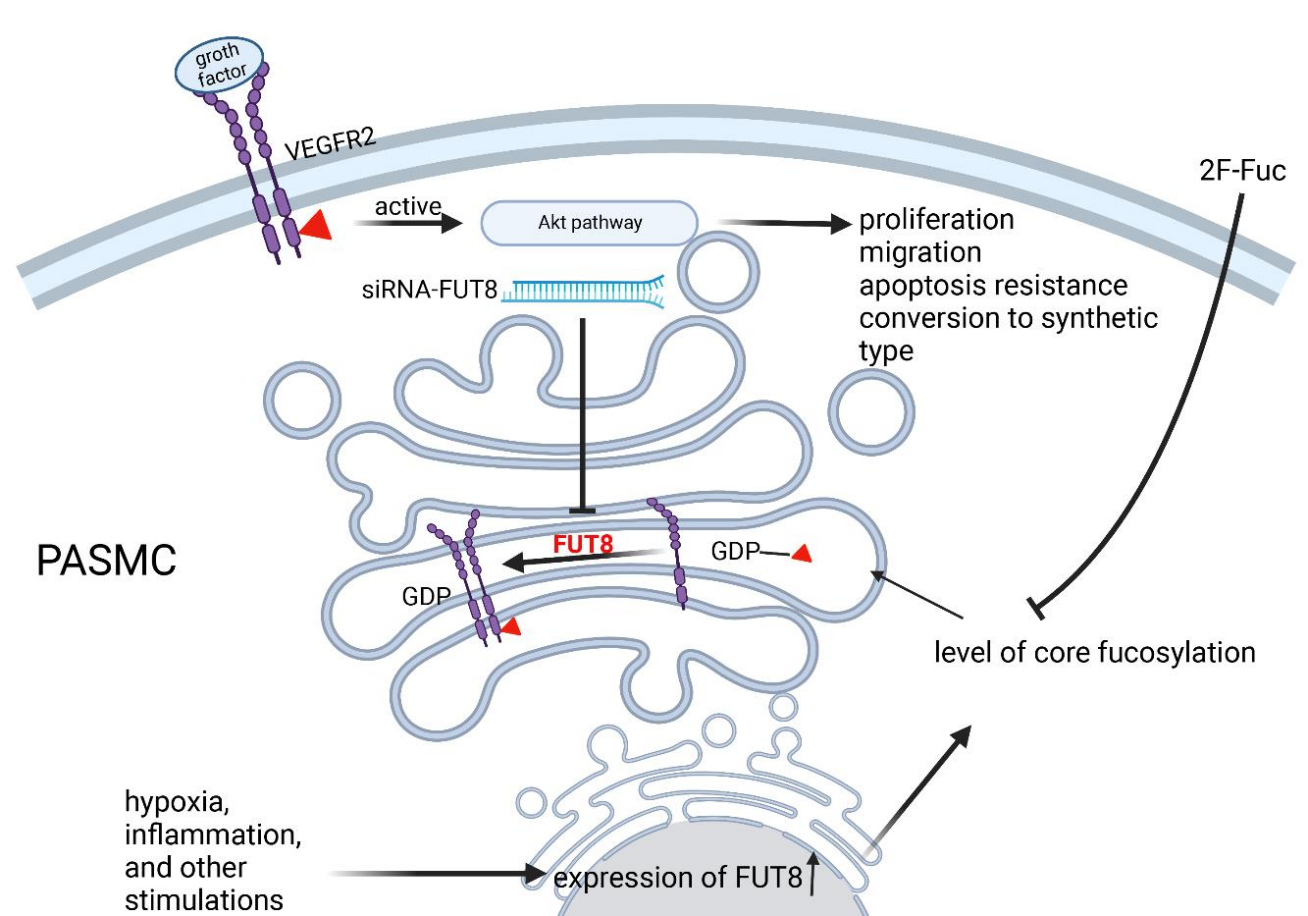
Model diagram describing the effect of FUT8 and its regulated core fucosylation on PASMCs. FUT8 expression may be increased by the stimulation of hypoxia, inflammation, or growth factors in PASMCs. Core fucosylation of whole cell proteins, including VEGFR2, is increased, which promotes activation of the AKT pathway, thereby promoting cell proliferation and migration, apoptotic resistance, and transformation to a synthetic phenotype, ultimately facilitating the process of vascular remodeling in PAH.

## Conclusions

FUT8 promotes PAH progression by upregulating core fucosylation levels and activating the AKT pathway. These findings demonstrate the relationship between core fucosylation and PAH and provide a novel avenue for the clinical diagnosis and treatment of PAH (Fig. 7).
